# Rectal buttonhole tear during parturition: A case report and literature review

**DOI:** 10.1186/s12884-026-08680-7

**Published:** 2026-01-31

**Authors:** Ye Tian, Lu Li

**Affiliations:** Central hospital affiliated to shandong first medical university, Jinan, Shandong China

**Keywords:** Rectal buttonhole tear, Rectovaginal septum, Isolated rectal tear, Delivery, Labor, Parturition

## Abstract

**Background:**

Isolated rectal tears with an intact sphincter lesion are an extremely rare clinical condition during parturition and are often referred to as a“buttonhole tear”.Currently, there is no established protocol for its management.The purpose of this study is to review the published literature of rectal buttonhole tears and describe a novel repair technique used in our case.

**Case presentation:**

All relevant articles were reviewed, including only case reports and case series. Our search identified 21 reported cases, comprising 10 normal vaginal deliveries, 7 operative ventouse deliveries, 4 forceps deliveries, and 1 vaginal breech delivery. All cases had an uneventful recovery except 1. Most authors recommended multilayer closure to reduce the risk of wound dehiscence and fistula formation.

In our case, a 23-year-old primigravida was admitted at 40+6 weeks of gestation. Her pregnancy was uncomplicated except for bacterial vaginosis. A healthy female newborn was delivered without complications. A rectovaginal examination revealed a rectal buttonhole tear with an intact anal sphincter. Following repair using a novel technique and postoperative management, the patient achieved an optimal recovery.

**Conclusions:**

Repair methods for rectal buttonhole tears vary. Given the rarity of this injury, the evidence base is limited. We propose a novel three-layer repair technique assisted by digital rectal support. Importantly, such lesions can be missed without a thorough post-delivery examination, potentially leading to delayed repair and long-term complications like rectovaginal fistula.

**Supplementary Information:**

The online version contains supplementary material available at 10.1186/s12884-026-08680-7.

## Background

A review of the literature reveals few cases reporting an obstetric isolated rectal tear with an undamaged anal sphincter [1]. This rarely encountered injury is likely underreported. There are no standardized recommendations for managing these injuries. The buttonhole rectal lesion is not included in the Sultan [2] or current American College of Obstetricians and Gynecologists(ACOG) [3]classifications due to its low incidence and distinct surgical management.However, an updated version of perineal trauma classification from the Royal College of Obstetricians and Gynaecologists (RCOG) incorporates the rectal buttonhole tear, which is not categorized as a fourth-degree tear by definition because it only involves the rectal mucosa while the anal sphincter complex remains intact [4].

## Methods and results

This study presents a review of all published case reports on isolated rectal buttonhole tears following vaginal delivery. A systematic literature search was conducted in PubMed using the following MeSH terms and keywords: “rectal buttonhole tear”,“rectovaginal septum”,“isolated rectal tear”, “delivery”, “labor” and “parturition”. All relevant articles were reviewed, including only case reports and case series. Cases involving concurrent fourth-degree perineal tears were excluded from this review. Data regarding patients’ basic information, intraoperative and postoperative management, and follow-up details were extracted from both our case and the identified publications.

A literature search initially identified four cases of isolated rectal tears during parturition [5, 6]. An additional seventeen cases were identified via hand searching [7, 18]. Therefore, 21 cases were included in our series (Table 1). Among the women described, there were 10 normal vaginal deliveries, 7 operative ventouse deliveries, 4 forceps deliveries, and 1 vaginal breech delivery. All women except 5 were primiparas. Episiotomy was performed in all instrumental deliveries except 1. A colorectal surgeon was consulted in 8 cases.

**Table 1:** Reported cases of buttonhole tear and their management

Paper	Age	Parity	Delivery	Injury	Repair	Postoperative	Follow-up
Chen,2023 [11]	26	0	normal vaginal delivery	0.5 cm buttonhole tear intact anal sphincter	by colorectal surgeon rectal mucosa:3 − 0 absorbable sutures rectovaginal septum:3 − 0 absorbable interrupted sutures vagina: routine closure	-	asymptomatic
Ngene,2023 [14]	26	1	normal vaginal delivery	5 cm buttonhole tear anal sphincter tear(3a)	by specialist obstetrician and gynaecologist rectal mucosa: continuous 2 − 0 Vicryl rectovaginal septum: interrupted PDS 3 − 0 vagina: continuous Vicryl 2 − 0	oral coamoxiclav for a week lactulose for 10 days paracetamol for 3 days	6 and 12 weeks follow up asymptomatic
Tunney, 2023 [15]	-	-	twin delivry with episiotomy ventouse and forceps	1.2 cm buttonhole tear intact anal sphincter	by consultant obstetrician rectal mucosa:3 − 0 PDS rectovaginal septum: four interrupted 2 − 0 Serapid sutures vagina: continuously with 2 − 0 Serapid	7 days of Gentamicin and Clindamycin cover Oral aperients for 14 days	6 weeks follow up asymptomatic
Tunney, 2023 [15]	-	-	singleton delivry with episiotomyforceps	1–2 cm buttonhole tear intact anal sphincter	by consultant obstetrician rectal mucosa:3 − 0 PDS rectovaginal septum: six separate single sutures of 3 − 0 PDS vagina: continuous locking manner using 2 − 0 Serapid	7 days of Gentamicin and Clindamycin cover Oral aperients for 14 days	6 weeks follow up asymptomatic
Djaković, 2022 [16]	31	0	normal vaginal delivery	4 cm buttonhole tear intact anal sphincter	-	broad spectrum antibiotics parenteral nutrition	-
Djaković, 2022 [16]	27	1	normal vaginal delivery with episiotomy	6 cm buttonhole tear intact anal sphincter	by colorectal surgeon colostoma rupture: synthetic, braided, absorbable 2 − 0 vicryl suture	broad spectrum antibiotics parenteral nutrition	2 months follow up colostomy closure
Awomolo, 2021 [5]	30	0	normal vaginal delivery	6 cm buttonhole tear intact anal sphincter	by colorectal surgeon rectal mucosa: continuous non-locking 3 − 0 biosyn rectovaginal septum: continuous non-locking 3 − 0 biosyn vagina: continuous locking 3 − 0 biosyn	cefazolin 3 g and ertapenem 1 g tylenol and ibuprofen	4 and 6 weeks asymptomatic
Habek,2021 [17]	27	1	ventouse delivery with episiotomy prolapsed whole right arm	10 cm buttonhole tear intact anal sphincter	rectal mucosa: Vicryl 4 − 0 rectovaginal septum: Vicryl 4 − 0 vagina: Vicryl 2 − 0	amoxiclav for 7 days metronidazole for 3 days pulpy fiber diet	6 weeks and 6 months follow up asymptomatic
Roper,2020 [6]	-	-	ventouse episiotomy	4–5 cm buttonhole tear	by colorectal surgeon 2-layer inverting 2–0 Vicryl	antibiotics Lactulose	3 months asymptomatic
Roper,2020 [6]	-	-	forceps episiotomy	buttonhole tear 3a tear	by obstetrician Interrupted 2 − 0 Vicryl rapide Knots in rectal lumen	antibiotics Lactulose	6 weeks asymptomatic
Roper,2020 [6]	-	-	Forceps episiotomy	3 cm buttonhole tear	by obstetric trainee re-sutured by consultant rectal mucosa: Interrupted 2 − 0 Vicryl Muscle: continuous 2 − 0 Vicryl Vaginal:2 − 0 Vicryl rapide	antibiotics lactulose	Wound breakdown secondary repair persistent fistula colostomy
Mercorio, 2020 [7]	29	0	normal vaginal delivery	4 cm buttonhole tear intact anal sphincter	rectal mucosa: interrupted adsorbable Vicryl 3 − 0 rectovaginal septum: Dexon 2 − 0 vagina: continuous unlocked Vicryl 3 − 0	cefalexin 1 g metronidazole 500 mg lactulose low fiber + high fluid diet	asymptomatic
Menzlova, 2014 [8]	32	0	normal vaginal delivery	1.5 cm buttonhole tear intact anal sphincter	by colorectal surgeon rectal mucosa(2 layers): absorbable sutures rectovaginal septum: no data vagina: routine closure	cefuroxime and metronidazole low fiber diet lactulose	14 days,3 months and 1 year asymptomatic
Vergers-Spooren,2011 [13]	29	0	breech delivery episiotomy	2–3 cm buttonhole tear intact anal sphincter	rectal mucosa: interrupted Monocryl 4 − 0 sutures rectovaginal septum: interrupted Monocryl 4 − 0 sutures vagina: continuous Vicryl 2 − 0	amoxycilline and augmentin magnesiumoxide	6 weeks and 3 months asymptomatic
Shaaban, 2008 [9]	32	0	ventouse	4 cm buttonhole tear intact anal sphincter	by consultant rectal mucosa: interrupted Vicryl sutures with knots in the rectal lumen vagina: continuous Vicryl sutures	metronidazole 0.5 g + cefuroxime 1.5 g fluids lactulose	6 weeks asymptomatic
Thirumagal,2007 [12]	37	1	normal vaginal	6 cm buttonhole tear intact anal sphincter	by colorectal surgeon rectal mucosa and muscularis: continuous Vicryl 1 − 0 vagina: Vicryl Rapide 2 − 0	antibiotics laxative	3 months asymptomatic
H.Byrne, 2006 [18]	38	0	ventouse delivery with episiotomy	5 cm buttonhole tear intact anal sphincter	by colorectal surgeon rectal mucosa: polyglactin 0 vagina: polyglactin 0	erythromycin metronidazole aperients low fibre diet	6 weeks follow up asymptomatic
Morrel, 1996 [10]	44	1	ventouse episiotomy	4 cm buttonhole tear intact anal sphincter	rectal mucosa: atraumatic inverting sutures vagina: routine closure	antibiotics laxative	asymptomatic
Morrel, 1996 [10]	29	0	ventouse episiotomy	4 cm buttonhole tear intact anal sphincter	rectal mucosa: inverting sutures vagina: routine closure	antibiotics laxative	asymptomatic
Morrel, 1996 [10]	27	0	normal vaginal	5 cm buttonhole tear intact anal sphincter	rectal mucosa: inverting sutures vagina: routine closure	antibiotics laxative	asymptomatic
Morrel, 1996 [10]	31	0	normal vaginal	3 cm buttonhole tear intact anal sphincter	rectal mucosa: continous inverting sutures	antibiotics laxative	asymptomatic

### Case presentation

A 23-year-old primigravida was admitted in spontaneous labour at 40 + 6 weeks of gestation. Her pregnancy was uncomplicated, except for bacterial vaginosis diagnosed at 40 + 5 weeks, for which she received oral metronidazole. The first stage of labor progressed satisfactorily with regular uterine contractions. When cervical dilation reached 10 cm, the delivery was managed by a specialist obstetrician. In the second stage, the perineum was excessively edematous, rigid and inflamed; therefore, a lateral episiotomy was performed at crowning. Bilateral sides of pudendal block anesthesia was administered before the episiotomy. The fetal head was delivered with perineal support, and the third stage of labour was completed uneventfully. A female newborn weighing 3000 g was born with normal Apgar scores.

The tear was not identified immediately after delivery. A rectovaginal examination performed after episiotomy suturing revealed a longitudinal rectovaginal “buttonhole tear” measuring 2 cm in length, with clean and smooth edges, located midline on the vaginal aspect of the episiotomy. Although the rectal mucosa was visible transvaginally, confirming the full-thickness nature of the tear, both the internal and external anal sphincters were intact. The distal end was approximately 3 cm proximal to the anus. Informed consent was obtained from the patient.

Surgical repair was performed in the delivery room by the specialist obstetrician. The surgical procedure included removal of the episiotomy sutures to fully expose the buttonhole tear. Repair was conducted under epidural anesthesia with adequate exposure, assistant support, optimal visualization, and thorough cleaning with saline solution followed by disinfection.

**Fig. 1:**
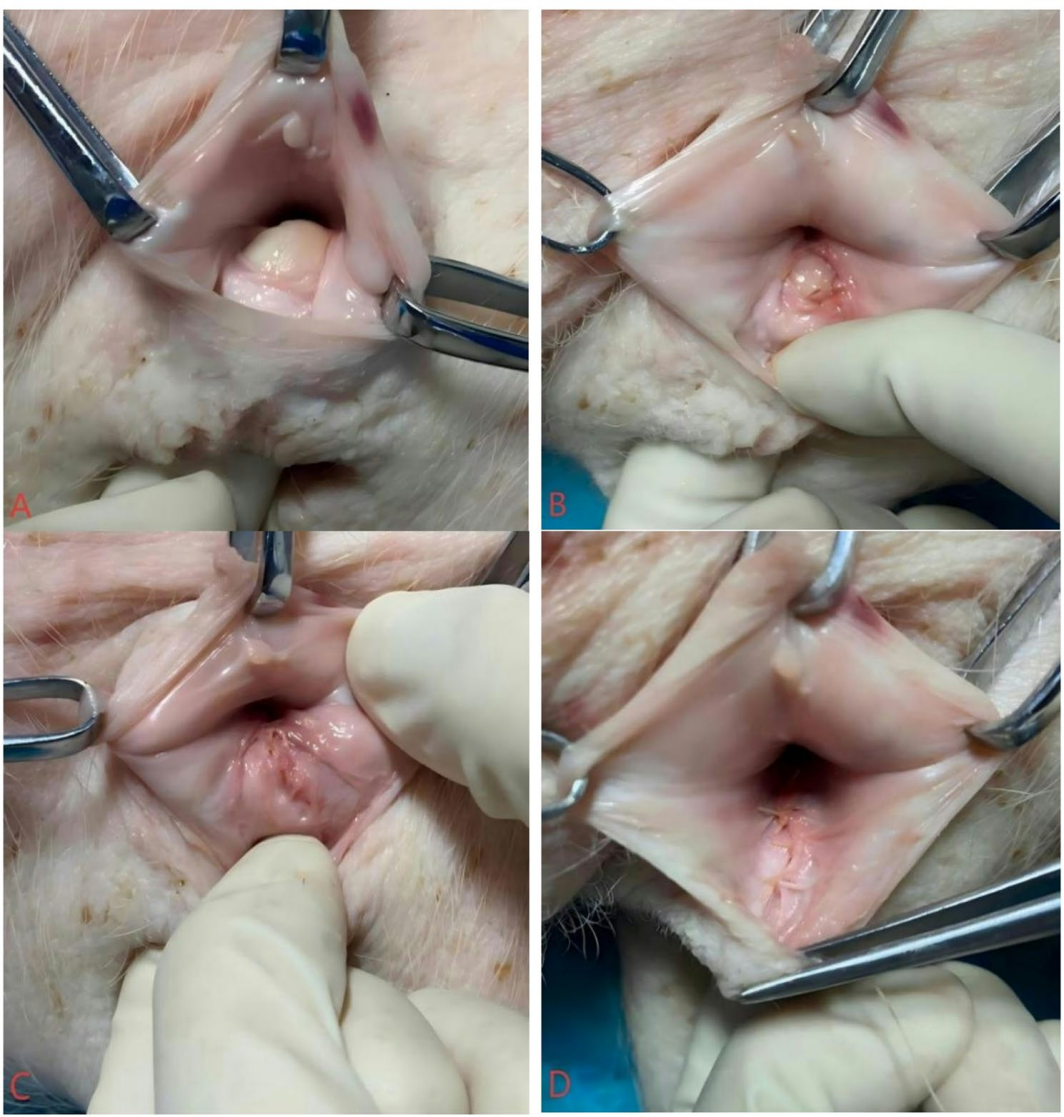
**A** Buttonhole tear prior to repair. **B** The sutured rectal mucosa. **C** The sutured rectovaginal septum. **D** The sutured vaginal mucosa

The assistant inserted an index finger into the rectal lumen and gently elevated the lesion, allowing full identification of the apex and distal end. The rectal mucosa was sutured with interrupted absorbable 3 − 0 Vicryl sutures. The repair was initiated at the apex of the rectal laceration, with the knots tied on the vaginal side to avoid mucosal penetration. Subsequently, the rectovaginal septum was repaired with interrupted absorbable 2 − 0 Vicryl sutures. Finally, the vaginal skin and episiotomy were routinely sutured with running absorbable 2 − 0 Vicryl sutures. No colostomy was performed. The suturing steps are demonstrated using a pig model simulating human obstetric buttonhole laceration repair. Figure 1A shows the laceration before repair. Figure 1B shows the sutured rectal mucosa. Figure 1C shows the sutured rectovaginal septum. Figure 1D shows the repaired vaginal mucosa.

The postoperative course was uncomplicated. The patient received empirical intravenous antibiotics for 4 days(cephalexin sodium 2 g three times daily and metronidazole 500 mg three times daily to cover anaerobic bacteria). Bowel movements were regulated with a low-fiber diet and lactulose for 4 days. She was discharged on postoperative day 3 after completing the intravenous antibiotic course, with clear instructions for a 6-week outpatient follow-up, and no symptoms of fecal incontinence or foul discharge were reported by the patient at discharge. Examination confirmed complete healing of the laceration and episiotomy, with no complications.

## Discussion

Correct identification and primary repair of buttonhole tears without colostomy are crucial. In our case, repair was successfully performed by the obstetric team without colorectal surgical consultation.The decision to proceed without consulting a colorectal surgeon was based on the specialist obstetrician’s experience and the isolated nature of the tear. No standardized technique exists for this injury. Based on recommendations by Roper et al. [6], we propose a feasible suture method involving an assistant who inserts an index finger into the rectal lumen to provide support and facilitate exploration, ensuring clear identification of the mucosal apex and distal ends. The surgeon then uses absorbable sutures to approximate the mucosal edges for optimal healing.However, a potential disadvantage of the digital assistance technique is the risk of needle-stick injury to the assistant. The use of a mounted swab or other instrument could be considered as an alternative to minimize this risk.

When repairing the rectal mucosa, knots can be placed in the rectal lumen or on the vaginal side [19]. The tear forms a “deep hole” extending from the vaginal to the rectal side, making suturing with knots in the rectal lumen challenging. Among reviewed cases, only two described knot placement in the rectal lumen [6, 9]. Therefore, we advocate repairing the rectal mucosa with knots tied on the vaginal side, which achieved an excellent outcome in our case.

The limited number of reported cases describes both two layer and three layer closures, while some reports even describe a four layer approach for enhanced strength [8]. Similarly, we recommend a three layer approach. The rectovaginal septum (rectovaginal fascia) should be clearly identified and repaired separately to enhance mid-layer strength and reduce fistula risk [6, 20].

Suture selection for approximating the laceration varied among reports. Vicryl (Polyglactin) was the most commonly used suture in most case series, as it causes less rectal irritation than polydiaxanone (PDS) suture. In our case, consistent with others, we used Vicryl for the rectal mucosa, rectovaginal septum, and vaginal wall. Based on our experience, we recommend interrupted Vicryl 3 − 0 sutures for the rectal mucosa, interrupted Vicryl 2 − 0 sutures for the rectovaginal septum, and continuous Vicryl 2 − 0 sutures for the vaginal skin.

Risk factors for perineal trauma include nulliparity, instrumental delivery, increasing age of the mother, high infant weight, perineal edema and prolonged second stage of labour [21, 22]. In our case, the baby was delivered easily before the inflamed and edematous perineal tissues could adapt elastically. Perineal edema and rapid fetal descent were likely contributing factors to the buttonhole tear.

This type of injury may occur more frequently than reported. A thorough digital rectal examination of the birth canal after placental delivery is necessary to detect potential buttonhole tears [4, 23, 24]. In our case, the tear was identified only after episiotomy suturing, highlighting that such lesions can be missed without careful vaginal and rectal examination prior to suturing. This can have a devastating impact on the social and sexual relationships of women.If missed, this can lead to devastating long-term consequences, including rectovaginal fistula, which severely impacts the social well-being, psychological state, and sexual relationships of affected women.Notably, among all reviewed cases, no colostomy was performed except in one case requiring secondary repair [6]. Most women were asymptomatic after primary repair, with satisfactory outcomes.

Our patient was 35 weeks pregnant again at the time of manuscript submission. Jordan et al. [25] described a protocol for determining the mode of delivery subsequent to OASIs. As there are no clear recommendations for delivery following a buttonhole tear, we will select an appropriate mode based on her preferences, rectal function, tissue integrity, and fetal weight.

## Conclusions

This report aims to raise awareness of rectal buttonhole tears and discuss their management. Key considerations include adequate anesthesia, optimal visualization, good exposure, experienced surgical technique, teamwork, and thorough cleaning/disinfection of the surgical field. If missed or inadequately repaired, a rectovaginal fistula may develop. Performing a rectal examination prior to perineal suturing is essential to identify occult damage. Further prospective studies with larger sample sizes are needed to validate the efficacy of the proposed technique.

## Supplementary Information

Supplementary Material 1.

Supplementary Material 2.

Supplementary Material 3.

Supplementary Material 4.

## Data Availability

No datasets were generated or analysed during the current study.
