# Supplementary material for: Rectal buttonhole tear during parturition: A case report and literature review
Source: BMC Pregnancy Childbirth. 2026 Jan 31;26:214. doi: 10.1186/s12884-026-08680-7 (PMC12952138; doi:10.1186/s12884-026-08680-7)
Supplement: Supplementary file 1 — Supplementary Material 1. [file 12884_2026_8680_MOESM1_ESM.docx]

Table1:Reported cases of buttonhole tear and their management

| Paper | Age | Parity | Delivery | Injury | Repair | Postoperative | Follow-up |
| --- | --- | --- | --- | --- | --- | --- | --- |
| Chen,2023^10^ | 26 | 0 | normal vaginal delivery | 0.5cm buttonhole tear intact anal sphincter | by colorectal surgeon rectal mucosa:3-0 absorbable sutures rectovaginal septum:3-0 absorbable interrupted sutures vagina：routine closure | - | asymptomatic |
| Awomolo,2021^4^ | 30 | 0 | normal vaginal delivery | 6cm buttonhole tear intact anal sphincter | by colorectal surgeon  rectal mucosa:continuous non-locking 3-0 biosyn rectovaginal septum:continuous non-locking 3-0 biosyn vagina：continuous locking 3-0 biosyn | cefazolin 3g and ertapenem 1g  tylenol and ibuprofen | 4 and 6 weeks asymptomatic |
| Roper,2020^5^ | - | - | ventouse  episiotomy | 4-5cm buttonhole tear | by colorectal surgeon  2-layer inverting 2–0 Vicryl | antibiotics  Lactulose | 3 months asymptomatic |
| Roper,2020^5^ | - | - | forceps  episiotomy | buttonhole tear  3a tear | by obstetrician  Interrupted 2-0 Vicryl rapide  Knots in rectal lumen | antibiotics  Lactulose | 6 weeks asymptomatic |
| Roper,2020^5^ | - | - | Forceps  episiotomy | 3cm buttonhole tear | by obstetric trainee  re-sutured by consultant  rectal mucosa:Interrupted 2-0 Vicryl  Muscle:continuous 2-0 Vicryl  Vaginal:2-0 Vicryl rapide | antibiotics  lactulose | Wound breakdown  secondary repair  persistent fistula  colostomy |
| Mercorio,  2020^6^ | 29 | 0 | normal vaginal delivery | 4cm buttonhole tear intact anal sphincter | rectal mucosa:interrupted adsorbable Vicryl 3-0 rectovaginal septum:Dexon 2-0 vagina：continuous unlocked Vicryl 3-0 | cefalexin 1 g metronidazole 500 mg lactulose low fiber+high fluid diet | asymptomatic |
| Menzlova，  2014^7^ | 32 | 0 | normal vaginal delivery | 1.5cm buttonhole tear intact anal sphincter | by colorectal surgeon rectal mucosa(2 layers):absorbable sutures rectovaginal septum:no data vagina:routine closure | cefuroxime and metronidazole low fiber diet lactulose | 14 days,3 months and 1 year asymptomatic |
| Vergers-Spooren,2011^12^ | 29 | 0 | breech delivery episiotomy | 2-3cm buttonhole tear intact anal sphincter | rectal mucosa:interrupted Monocryl 4-0 sutures rectovaginal septum:interrupted Monocryl 4-0 sutures vagina:continuous Vicryl 2-0 | amoxycilline and augmentin magnesiumoxide | 6 weeks and 3 months asymptomatic |
| Shaaban,2008^8^ | 32 | 0 | ventouse | 4cm buttonhole tear intact anal sphincter | by consultant  rectal mucosa:interrupted Vicryl sutures with knots in the rectal lumen vagina:continuous Vicryl sutures | metronidazole 0.5g+cefuroxime 1.5g  fluids  lactulose | 6 weeks asymptomatic |
| Thirumagal,2007^11^ | 37 | 1 | normal vaginal | 6cm buttonhole tear intact anal sphincter | by colorectal surgeon  rectal mucosa and muscularis: continuous Vicryl 1-0 vagina：Vicryl Rapide 2-0 | antibiotics laxative | 3 months asymptomatic |
| Morrel,1996^9^ | 44 | 1 | ventouse episiotomy | 4cm buttonhole tear intact anal sphincter | rectal mucosa:atraumatic inverting sutures vagina:routine closure | antibiotics laxative | asymptomatic |
| Morrel,1996^9^ | 29 | 0 | ventouse episiotomy | 4cm buttonhole tear intact anal sphincter | rectal mucosa:inverting sutures vagina:routine closure | antibiotics laxative | asymptomatic |
| Morrel,1996^9^ | 27 | 0 | normal vaginal | 5cm buttonhole tear intact anal sphincter | rectal mucosa:inverting sutures vagina:routine closure | antibiotics laxative | asymptomatic |
| Morrel,1996^9^ | 31 | 0 | normal vaginal | 3cm buttonhole tear intact anal sphincter | rectal mucosa:continous inverting sutures | antibiotics laxative | asymptomatic |
