# Supplementary material for: Rectal buttonhole tear during parturition: A case report and literature review
Source: BMC Pregnancy Childbirth. 2026 Jan 31;26:214. doi: 10.1186/s12884-026-08680-7 (PMC12952138; doi:10.1186/s12884-026-08680-7)
Supplement: Supplementary file 2 — Supplementary Material 2. [file 12884_2026_8680_MOESM2_ESM.docx]

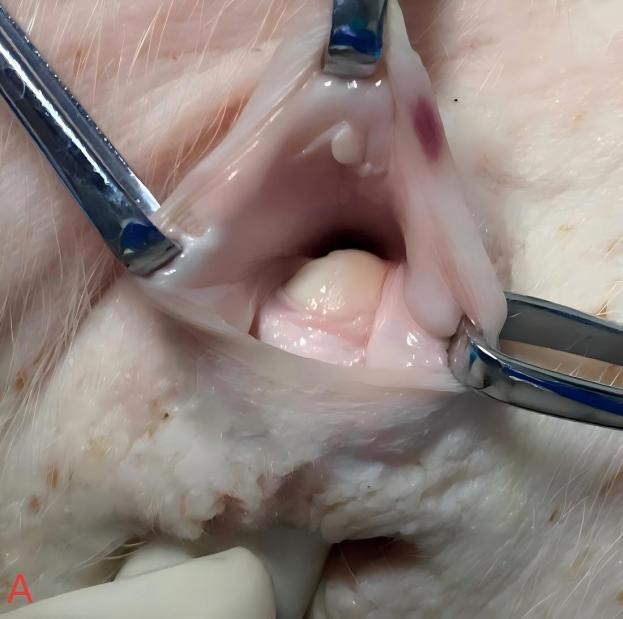

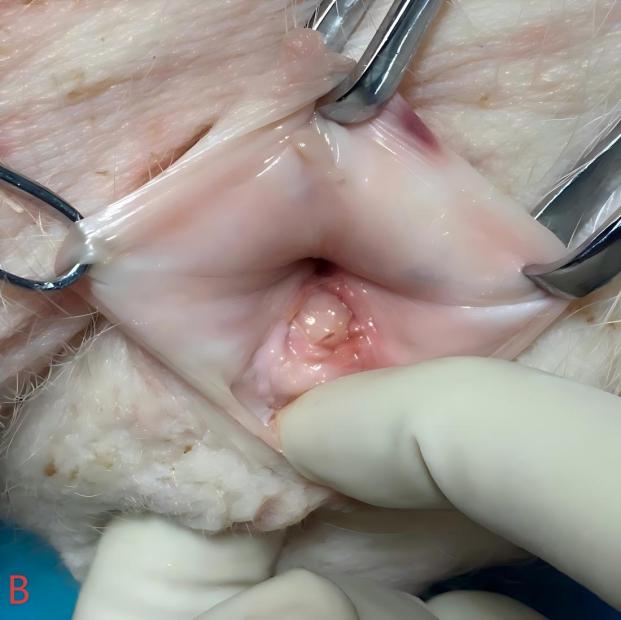

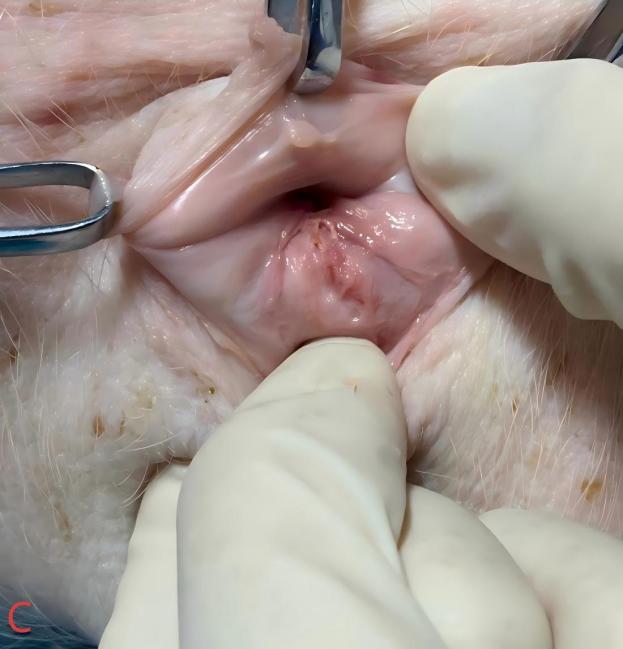

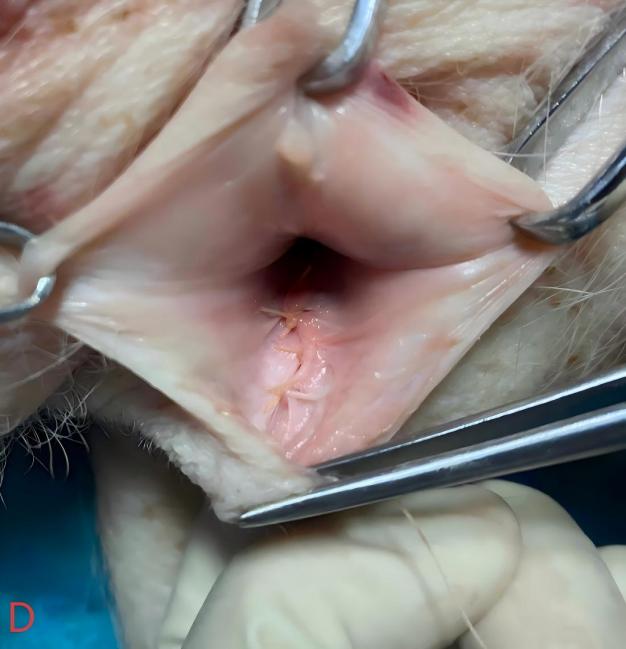


Fig.1 A:buttonhole tear prior to repair B:the sutured rectal mucosa

C:the sutured rectovaginal septum D:the sutured vaginal mucosa
