# Supplementary material for: Rectal buttonhole tear during parturition: A case report and literature review
Source: BMC Pregnancy Childbirth. 2026 Jan 31;26:214. doi: 10.1186/s12884-026-08680-7 (PMC12952138; doi:10.1186/s12884-026-08680-7)
Supplement: Supplementary file 3 — Supplementary Material 3. [file 12884_2026_8680_MOESM3_ESM.docx]

Subject: Submission of Manuscript for Publication in BMC Pregnancy and Childbirth

Dear Editors and Referees,

On behalf of all co-authors, I am pleased to submit our article entitled“Rectal Buttonhole Tear During Parturition and a Novel Repair Technique: A Case Series and Literature Review”authored by Tian Ye and Li Lu for consideration as a case report paper in the “BMC Pregnancy and Childbirth”.

The reasons for selecting the “BMC Pregnancy and Childbirth” are as follows:

1.This manuscript reports an extremely rare clinical condition during parturition, with limited data available in literature;

2.As your journal prioritizes clinical presentations, diagnoses and/or management of new and emerging diseases, our focus on a novel repair technique for this underreported condition aligns closely with your Aims and Scope;

3.Current management of rectal buttonhole tears lacks an established protocol, and your journal’s readership would benefit from our practical recommendations;

4.Correct identification and primary repair of this tear is clinically challenging, and our findings can inform evidence-based practice for your journal’s audience.​

In this work, we review the published literature of rectal buttonhole tears and describe a novel repair technique used in our case. Notably, such lesions can be missed if thorough vaginal and rectal examination is not performed as a standard procedure following delivery, which may lead to delayed repair and long-term complications such as rectovaginal fistula.

The major findings and remarkable novelties of the present work can be summarized as follows:

1.We propose a novel repair technique for this rare laceration: an assistant inserts their index finger into the rectal lumen to provide support and perform exploration, which can clearly identify the apex and distal ends of the mucosa.Subsequently, the surgeon uses absorbable sutures to reapproximate the mucosal edges and ensure proper healing.

2.We outline key clinical considerations for managing these lacerations, including adequate anesthesia and visualization, good exposure, experienced surgical techniques, teamwork, and complete cleaning and disinfection of surgical field;

The manuscript is original, has not been published previously, and is not under consideration for publication elsewhere.The participating patient provided written informed consent.All authors have read and approved the final version of the manuscript, meet the ICMJE criteria for authorship, and declare no conflicts of interest related to this work.

We believe this manuscript is well-suited to the readership of the BMC Pregnancy and Childbirth. If you require any additional materials, Please contact the corresponding author for any inquiries.

We would greatly appreciate your consideration of our manuscript.

With best wishes.

Corresponding Author: Li Lu

1. mail:413505122@qq.com

ORCID: 0009000488939940
