# Supplementary material for: Rectal buttonhole tear during parturition: A case report and literature review
Source: BMC Pregnancy Childbirth. 2026 Jan 31;26:214. doi: 10.1186/s12884-026-08680-7 (PMC12952138; doi:10.1186/s12884-026-08680-7)
Supplement: Supplementary file 4 — Supplementary Material 4. [file 12884_2026_8680_MOESM4_ESM.docx]

**Category of pape**r: Case Report

**Article title**: Rectal Buttonhole Tear During Parturition and a Novel Repair Technique: A Case Series and Literature Review

**Author names**:

Tian Ye:obstetrics, Shandong First Medical University Affiliated Jinan Central Hospital, jinan, 250014, china

E-mail:judy20080801@163.com

ORCID:0009000228354823

**Corresponding author:**

Li Lu:obstetrics, Shandong First Medical University Affiliated Jinan Central Hospital, jinan, 250014, china

1. mail:413505122@qq.com

ORCID:0009000488939940

Mailing address:Obstetrics Ward 2, Building 7, No.105 Jiefang Road, Lixia District, Jinan City, Shandong Province, China

Telephone numbers:+86 19153122281

**Conflict of Interest Statement:**

All authors declare that they have no known competing financial interests or personal relationships that could have appeared to influence the work reported in this manuscript.
